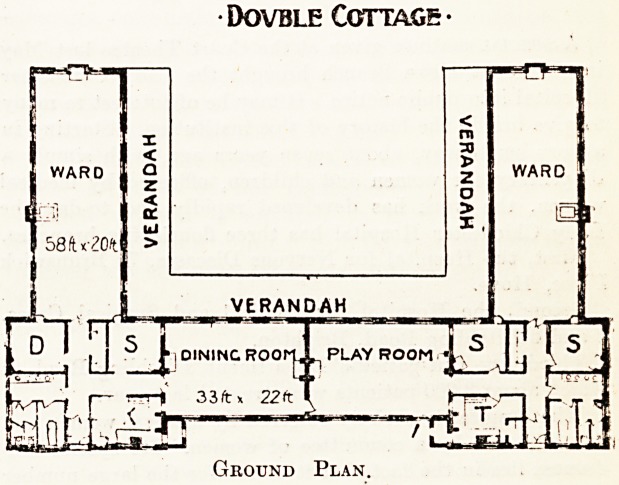# Queen Mary's Hospital for Children, Carshalton

**Published:** 1913-07-26

**Authors:** 


### QUEEN MARY'S HOSPITAL FOR CHILDREN, CARSHALTON.

Queen Mary's Hospital for Sick Children, Carshalton, is unique among children's hospitals. Established as it was under the Metropolitan Asylums Board, it is the first public hospital to receive Royal recognition of the value of its work by obtaining the Queen's permission to use her name in its title. It is the largest children's hospital in this country, and possibly in the world. It is built on a plan which is different from that which the voluntary hospitals have adopted. Its size and site come more near to that often discussed scheme of the future, the hospital city, than perhaps any other yet in existence. Thanks to the courtesy of the Medical Superintendent, Dr. Gordon Pugh, our Commissioner was able to make a tour of the institution, and to learn from him something of its work and of the way in which its effective administration has been formed to meet the peculiar problems necessitated by its design.

"I daresay you already know," Dr. Gordon Pugh began, "that this is the latest institution of magnitude established by the Metropolitan Asylums Board. As you are probably aware, the Board consists of representatives of the London Boards of Guardians and of members appointed by the Local Government Board. Established in 1861 to deal with the problems of the sick and infirm of the Metropolitan Unions, it is divided into three committees, which control respectively its infectious diseases hospitals, its mental hospitals, and its children's hospitals. What is now known as Queen Mary's Hospital was originally intended as a convalescent fever hospital. But the smallpox epidemic of 1902 led to an extension of the Board's accommodation at Dartford, which set free these buildings for other purposes."

"When did it start work?"

"It was opened by Mr. John Burns in January 1909, and has at present accommodation for 850 children. When it was decided that the institution was to be used for a great children's hospital, it was originally christened The Children's Infirmary. This name, however, was felt by many to be quite inadequate to express the scope and work of the institution, and Mr. Burns, having been approached by the Board, obtained her Majesty's gracious permission to have it named after her. The site, which forms the highest point in the neighbourhood—from which, for instance, Harrow can be seen on a clear day—consists of 136 acres, and the fact that it was originally intended for infectious cases led to a series of cottage blocks being built in contradistinction to the pavilion system, which is more generally seen in children's hospitals at present. These cottage blocks, each of which, as the accompanying illustration shows, is a one-storeyed building, built round three sides of a square, with a tar-paving space or courtyard between, are dotted over the site in the following manner:—At the north and south extremities of the site are respectively the administration block and the block reserved for surgical cases. A straight road, some 400 yards long, joins the two buildings. On either side of this main thoroughfare three streets branch off, each of which contains four cottage blocks of the design I have just described. You get, therefore, a central road, with a large block at each end, and twelve cottage blocks

Ground Plan, Queen Mary's Hospital.

on either side of it. Of these, four cottages in one street are divided into curtain-cubicles for the domestic staff. In addition to two large nursing homes near the administration block a small staff home is attached to each street, and contains the house sister's quarters and the bedrooms of some of the nursing staff. The architects are Messrs. Treadwell and Martin. Including engineering and road-making, about £233,000 was the cost of building."

"I notice that you have no covered ways."

"There is only one, that between the two portions of the surgical block. But for the rest great care was taken and much expense incurred on our tarmac roads, which have a hard, smooth surface, and consequently dry rapidly. The staff are provided with cloaks and goloshes, and we find that answers perfectly well. Now let us go over a typical cottage block. Although they are not all used for the same purpose, each indeed being devoted to some special use, and thus allowing the children to be classified according to their diseases, they are all of similar design."

Dr. Pugh then led the way into the cottage block devoted to cases of joint disease in girls. As the plan shows, the two arms of the cottage block are the wards, each designed for sixteen patients, while the space which joins the two arms is divided between a dining room and a playroom. In addition are lavatory accommodation, four separation rooms of two beds each, and a ward kitchen, where breakfasts and suppers can be cooked, or the luncheons warmed on their arrival from the main kitchen. Noticing that the wooden floor of the ward was of a yellow colour, more like satinwood than oak or teak, I asked what wood it was.

"Maple," said Dr. Pugh. "It is a very hard wood, almost as hard as teak, and superior to it from the fact that it does not show the marks of footprints. The tables in the dining room are birch, and, being English instead of French polished—that is, having beeswax and turpentine only put on them, without any varnish—they do not show any marks from hot dishes or spilt water, and can readily be kept polished. Another point of detail in the ward furniture is the castor attached to the bed-legs. Instead of having an arm of its own which is arbitrarily attached at an angle to the foot of the bed-leg, with the result that the castor periodically jambs and will not move easily in a sudden change of direction, this castor is fixed at the bottom of a straight pin with a conical end which runs for some inches up the centre of the hollowed bed-leg, and revolves in any direction without friction. Running round the base of the cone, at the inside of the bed-leg, is a groove which receives the inner end of a screw, and this prevents the castor from falling off when the end of the bed is lifted.

"The most important feature of each cottage block is, however, the verandah, for by its means the accommodation of each block is nearly doubled. Originally the verandah was fitted only along the south face of the block courtyard. It has been extended, however, in several instances round the three sides, and consists of a glass roof with metal stays and supports. The distinctive feature of the verandahs is, however, the shelter that can be pulled down in wet weather. Various types were tried, but none proved satisfactory until we hit on the device of the ordinary shop-window shutter. This rolls up like the lid of an American writing-desk, and by the action of a spring is able to remain at any level from the verandah roof to which it may be lowered. The verandah is ten feet wide. Where these are in use the number of patients in each cottage block can be raised from forty to seventy. This increased figure, moreover, reduces the cost per bed considerably. The cost of each cottage block has been about £4,000, and of each verandah about £300. By doubling the number of patients, you halve of course the cost per bed."

"Is a verandah being added in every case?"

"We cannot do that at present, for the increased numbers of patients made possible by the verandahs demand an increased staff, and we have been, and still are, short of staff accommodation. Now three cottage blocks are given up entirely to the domestic staff, the wards of which are divided into curtained cubicles. Everything except the actual buildings is centralised as far as possible. When the cottage blocks were first designed for infectious cases, a local heating system was provided, because, of course, the number of cottage blocks in use would be constantly varying in number, and a central heating system would have been very wasteful. The moment, however, that it was decided to convert the buildings into a children's hospital—a hospital, in other words, likely always to be full—a central heating system became both economical and inevitable. Now, in spite of the extent of the site, hot water is pumped through the entire institution."

"Then as regards the staff?"

"I have no difficulty here in getting assistant medical officers. The opportunities for the specialised study of children's diseases are sufficiently great and varied to attract men who are keen on the work. There are five assistant medical officers, all of whom, to be eligible for a post here, must have held appointments in general hospitals. There is also a visiting dental surgeon. As regards the nurses, other institutions sometimes complain that it is harder than it was to obtain the right class of women, owing to the number of posts and professions now open to their sex. For example, the Bank of England now employs women clerks. We are very careful to accept applicants only from the class we want. Our nursing staff numbers 159, 92 of whom are probationers. We take on probationers at nineteen—strictly excluding the domestic servant class—and give them a three years' training with an examination for a certificate at the end of that period. Outside examiners—a physician and surgeon on the staff of a London general hospital, with special experience in the teaching of nurses—hold the examination, the passing of which gives a certificate, and the nurses are then of age to enter a general hospital, where the possession of a certificate here stands them in good stead. Some stop on and join our permanent staff, for the life is certainly a pleasant one, and the grounds comprise a full-size hockey ground, tennis and croquet lawns, and so on. There are for them too opportunities of specialising. For instance, we were, I think, the first institution in the country to start classes for probationers in the Swedish remedial exercises, massage, and allied treatments. In December 1910 a gymnasium was equipped with plinths, booms, ribstols, and other appliances for Swedish remedial exercises and massage, and a massage sister was appointed to carry out the treatment and also to instruct the nurses in it. The following fees are charged: probationers, £1 1s., to be returned on passing the examination of the Incorporated Society of Trained Masseuses. Other members of the nursing staff, £3 3s. The massage sister is required to have attended a two years' course, including training as a teacher. For tuberculous children the graduated labour system is in force among the boys, while the tuberculous girls have a graduated drill, which at its most elaborate point is a genuinely strenuous form of exercise."

"The administration over so many scattered buildings has been a difficult problem?"

"It was difficult at first to set it in working order. The system entails a sister in charge of each street, and a day and night staff for each block. The problem

Gymnasium.

of serving the food may seem at first sight to present a difficulty, but this is overcome by having a central kitchen, from which electric motors carry the meals to their respective destinations. This system answers very well. The time taken from the kitchen to the destination averages under ten minutes, a period as short as, if not shorter than, that often taken from an ordinary institutional kitchen to the ward. The electric motors are covered vans designed to carry 30 cwt., and are accompanied by two men who deliver the food, which is kept hot in jacketed bins which can be placed on the

The Verandah.

gas-stove in the block kitchen to be further warmed if necessary. The patients who take their meals on the balconies have their food served on hot-water plates."

Dr. Gordon Pugh, after a visit to the milk-testing and milk-pasteurising rooms, and the *x*-ray room, led the way to the kitchen, which, he explained, is in the charge of a certificated lady cook, who has gained the certificate of the National Training School of Cookery, known as the cook superintendent. She receives a salary of £60 per annum, with the emoluments of board, lodging, washing, and uniform, and enjoys the same status as that assigned to the ward sisters. Among her duties is included the instruction of the probationer-nurses in sick-room cookery. The post of cook-probationer has also been created, in which a newly certificated lady cook is enabled to acquire a knowledge of institutional cooking, receiving a salary of £30 per annum, with the same status in the institution as her immediate chief. A certificate of training in institutional cooking and the administration of a large kitchen is given at the end of twelve months.

"I want to draw your attention to one or two of our labour-saving devices," Dr. Pugh continued. "Here, for example, is a machine for making chipped potatoes, and next to it a 'Victoria' Carborundum Potato-Peeling Machine, made by the Imperial Machine Co., Cricklewood Lane. Here is an improvement at present unique, namely, a Berkel's Patent Slicing Machine, driven not by hand but by motor. Everything that is boned, whether hot or cold meat, is sliced here. It was because there was so much work to do that we decided to have it driven by motor. There are also special boilers for hams, which

·Dovble Cottage·

Ground Plan.

allow the bones to be removed and then compress the hams into their normal shape, with the net result that there is no waste in cooking or carving, and that practically the only weight lost is that of the bone itself. The mincing machine is also driven by motor. Most of the cooking is done by gas."

"Then as to the patients?"

"They range from three weeks to sixteen years of age, after which we neither accept nor keep them, and they come either from their homes through the recommendation of the district medical officers or, as in the majority of cases, from the Metropolitan infirmaries, while in addition we receive cases for operation from the Board's training-ship *Exmouth*. We also occasionally exchange cases with the Board's other institutions, especially Millfield House, Rustlington, Sussex, which is really for medical tubercular cases. The scope of the work here may be judged from the purposes of the various cottage blocks, each of which is a kind of special hospital. Many patients stay for a prolonged period, and educational instruction is provided by four certificated mistresses, empty wards being used temporarily as school-rooms. School-rooms and a recreation hall are to be built this summer. A shoemaker instructor is also engaged for instructing cripples in the art of cobbling. The patients' accommodation may be summarised as follows: In the surgical section there are three wards for cases requiring operation or special attention. One block of seventy beds for diseases of joints, requiring recumbency, in boys; a similar block for the same purpose on the girls' side; a block of seventy beds for diseases of the spine; a block for each sex for the treatment of patients who are able to get up, such as lateral curvature, club-foot, paralysis, tubercular glands, lupus, convalescent joint and spine cases, otorrhœa, etc. In the medical section there are three wards for marasmic babies and children suffering from debility after various diseases; a block of forty beds reserved for diseases of the heart, skin, and nervous system in girls; a block of seventy beds for a similar purpose on the boys' side; a block of four wards and four rooms reserved for isolation purposes. The sanatorium section consists on the girls' side of a verandah block of seventy beds for early cases and a similar block for more advanced cases; and on the boys' side of a block for cases not getting up all day; two blocks for those getting up all day, one being used as a dormitory for ninety boys, the other provides dining room, reading room and playrooms, with verandah accommodation for thirty beds."

"What do you think of the cottage block as a form of hospital architecture?"

"I think it is the form of the future. Under the verandah system there is great economy of space, and, as already explained, great economy in building when judged by the cost per bed. Also there seems to be little doubt that more and more will hospital patients generally be treated in the open air, and not in enclosed wards or pavilions. Site space, of course, is required. But we are showing here that the administrative problems may be overcome as satisfactorily as the engineering problems, for with all our miles of pipes we have had so far but little to complain of."

## Figures and Tables

**Figure f1:**
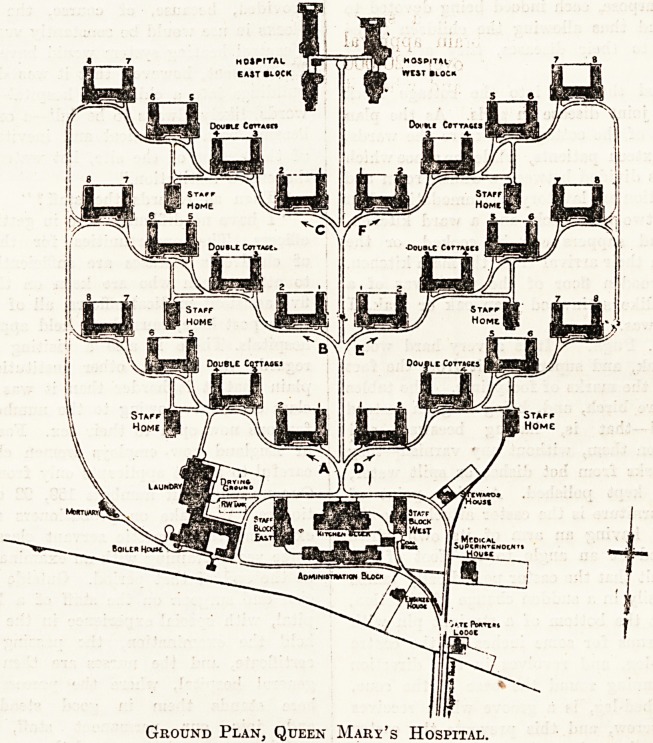


**Figure f2:**
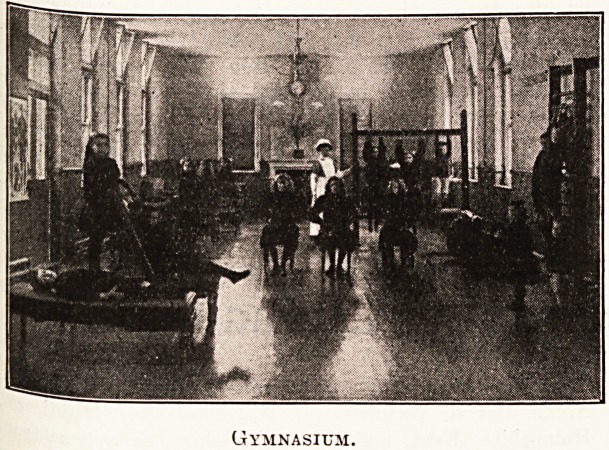


**Figure f3:**
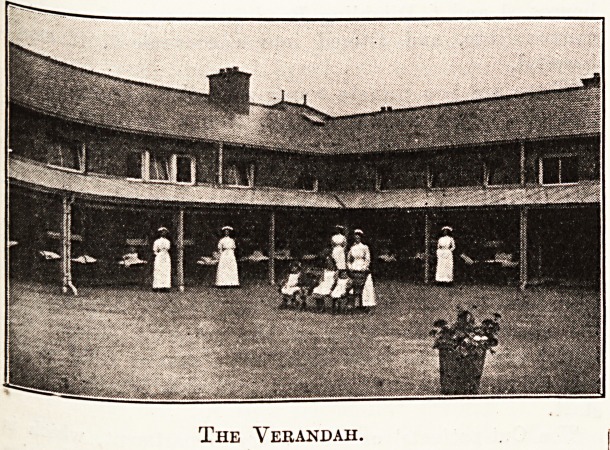


**Figure f4:**